# Targeted base editing in the plastid genome of *Arabidopsis thaliana*

**DOI:** 10.1038/s41477-021-00954-6

**Published:** 2021-07-01

**Authors:** Issei Nakazato, Miki Okuno, Hiroshi Yamamoto, Yoshiko Tamura, Takehiko Itoh, Toshiharu Shikanai, Hideki Takanashi, Nobuhiro Tsutsumi, Shin-ichi Arimura

**Affiliations:** 1 Laboratory of Plant Molecular Genetics, Graduate School of Agricultural and Life Sciences, The University of Tokyo, Tokyo, Japan; 2 School of Life Science and Technology, Tokyo Institute of Technology, Tokyo, Japan; 3 Division of Microbiology, Department of Infectious Medicine, Kurume University School of Medicine, Kurume, Japan; 4 Department of Botany, Graduate School of Science, Kyoto University, Kyoto, Japan

**Keywords:** Genetic engineering, Transgenic plants

## Abstract

Bacterial cytidine deaminase fused to the DNA binding domains of transcription activator-like effector nucleases was recently reported to transiently substitute a targeted C to a T in mitochondrial DNA of mammalian cultured cells^1^. We applied this system to targeted base editing in the *Arabidopsis thaliana* plastid genome. The targeted Cs were homoplasmically substituted to Ts in some plantlets of the T_1_ generation and the mutations were inherited by their offspring independently of their nuclear-introduced vectors.

## Abstract (web summary)

This study used bacterial cytidine deaminase fused to the DNA binding domains of transcription activator-like effector nucleases to enable targeted base editing in the *Arabidopsis thaliana* plastid genome, generating T_1_ plants with inheritable homoplasmic mutations.

## Main

Plastid genomes, which encode key genes for photosynthetic processes, including both light reactions and carbon assimilation, are potential targets of plant breeding. Plastid genetic transformation can now be used for only a limited number of species^2^ and is difficult even in the model plant *Arabidopsis*^3,4^. In addition, it requires insertion of a marker gene into the plastid genome^5^, so the created plants are regarded as genetically modified organisms (GMOs). Recently, cytidine deaminase (CD), which converts C to U to change G/C pairs to A/T pairs in double-stranded DNA, was successfully used for in vitro targeted base editing of mitochondrial DNAs in mammalian cultured cells^1^. Here, we applied this technology to edit targeted bases in three genes in the plastid genome in *Arabidopsis* plantlets, without leaving any foreign genes in either the plastid or nuclear genomes. Targeted single nucleotide substitutions are expected to be the best way to make desired single nucleotide polymorphisms (SNPs) without disturbing any other genes or regulatory regions in the plastid genomes of common crops or elite lines. For the targets, we selected three genes whose modifications would be expected to lead to observable effects; *16S* *rRNA*, whose modification was expected to confer resistance to an antibiotic and two genes whose modifications would lead to poor growth; *rpoC1*, which encodes a part of the DNA-directed RNA polymerase subunit beta′; and *psbA*, which encodes photosystem II (PSII) protein D1.

**Fig. 1:**
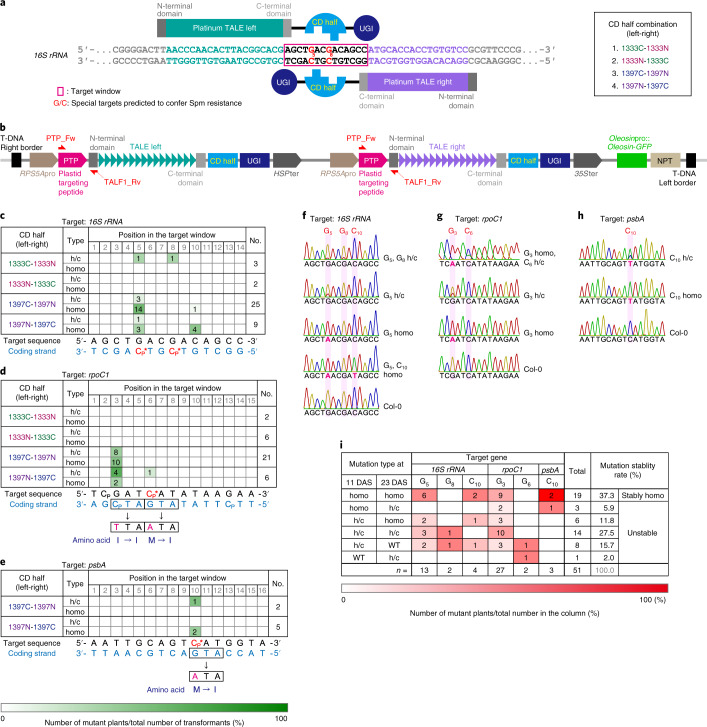
ptpTALECD for three plastid genes. **a**, A pair of pTALECD proteins and its targeting window (shown in a red rectangle) in *16S* *rRNA* gene and CD half combination. Substitution of the fifth and/or eighth C/G pairs (shown in red) with T/A pairs was predicted to confer Spm resistance. **b**, T-DNA region of the tandem expression vectors for ptpTALECD. **c**–**e**, Base-edited plant numbers, editing efficiencies shown in colour (bottom) and predicted amino acid substitutions in the three target windows of 23 DAS T_1_ plants (**c**, *16S rRNA*; **d**, *rpoC1*; **e**, *psbA*). **f**–**h**, Representative data from Sanger sequencing in the ptpTALECD targeting windows of 23 DAS T_1_ plants (**f**, *16S* *rRNA*; **g**, *rpoC1*; **h**, *psbA*). **i**, Transition of substitution frequency states at the targeted bases between 11 DAS and 23 DAS T_1_ plants. Abbreviations: h/c, heteroplasmically or chimaerically substituted; homo, homoplasmically substituted; Cp, preferential target cytosines; and C*, special target cytosines predicted to cause biological effects.

**Extended Data Fig. 1:**
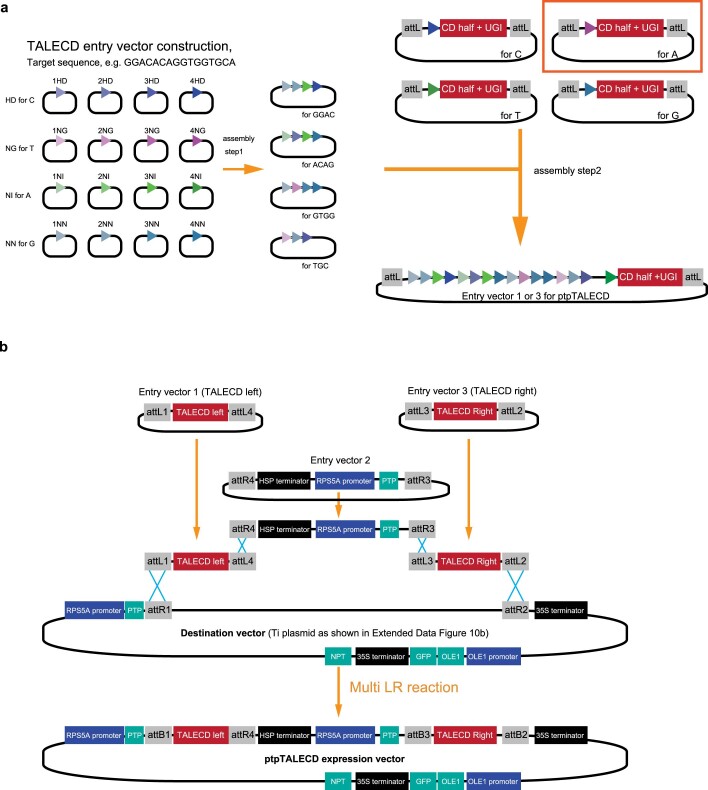
Schematic image of constructing plastid-targeting pTALECD (ptpTALECD) expression vectors. **a**, Assembly steps for constructing pTALECD ORFs. Platinum TALEN Kit was used but entry vectors for step2 were those generated as Extended Data Fig. 10a shows. **b**, The ptpTALECD expression vectors were constructed with LR Clonase™ II Plus enzyme (Thermo Fisher Scientific). NPT: kanamycin resistance gene.

**Extended Data Fig. 2:**
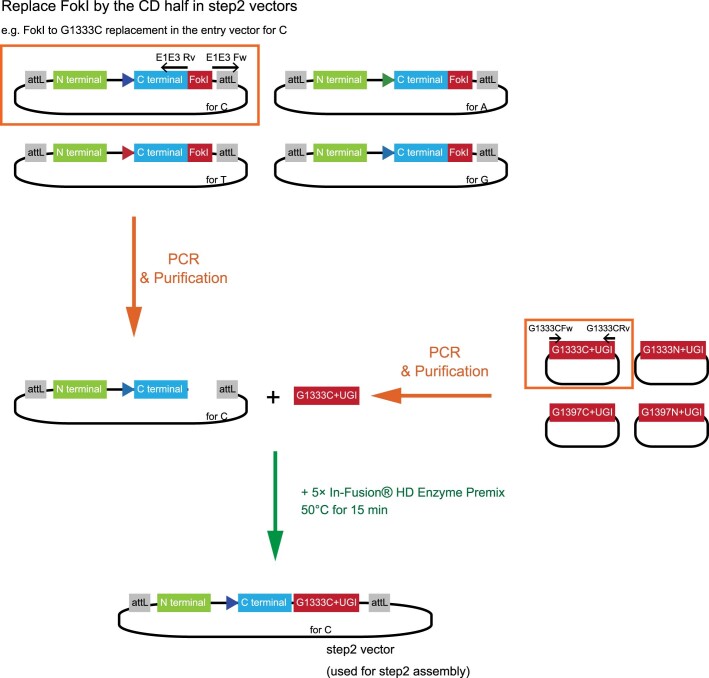
Replacement of the FokI coding sequence in entry vectors for step2 assembly by the cytidine deaminase (CD) half coding sequence. Artificially synthesized CD half coding sequence (Supplementary Table 4) and entry vectors for step2 assembly containing FokI coding sequence were amplified with primes corresponding to the template (Supplementary Table 5). The purified PCR products were mixed with 5× In-Fusion HD Cloning Enzyme Premix (TaKaRa) and incubated at 50 °C for 15 minutes.

As in the previous study^1^, the CD domain (163 amino acids (aa)) of *Burkholderia cenocepacia* DddA toxin (1,427 aa) was split at the 1,333th or 1,397th amino acid. Each of the amino terminal or carboxy terminal halves of the CD was linked to the C terminus of the DNA binding domain of the platinum TALEN^6^ (pTALECD; Fig. 1a). The N terminus of the pTALECD was linked to a plastid-targeting signal peptide (PTP) of *Arabidopsis thaliana* RecA1 protein (51 aa)^7,8^ (Fig. 1b), while the C terminus was linked to an uracil glycosylase inhibitor (UGI)^1,9^ to inhibit hydrolysis of the generated uracil (Fig. 1b). The nucleotide sequences of CD and UGI were optimized for *A. thaliana* codon usage. A pair of PTP-pTALECD-UGIs (ptpTALECDs) were expressed in a single plant transformation vector under control of efficient *RPS5A* promoters^10^ (Fig. 1b). We established a system to smoothly assemble the complicated tandem expression vectors of ptpTALECD for each target sequence on the Ti plasmid (Extended Data Fig. 1) by replacing the FokI in the vectors used in a previous study^11^ with CD-UGI (Extended Data Fig. 2). We introduced the vectors into the nucleus of *A. thaliana* by floral dipping^12^ and attempted to substitute C/G to T/A in *16S* *rRNA* (Fig. 1c), *rpoC1* (Fig. 1d) and *psbA* (Fig. 1e). Substitution of G_5_ and/or G_8_ in *16S* *rRNA* (highlighted in red in Fig. 1a) to A would confer spectinomycin (Spm) resistance (below)^13,14^ while substitutions of C_6_ in *rpoC1* and C_10_ in *psbA* to Ts would lead to changes in initiation codons from ATG (methionine) to ATA (isoleucine). As a result, accumulation of their coding proteins would decrease and mutants would grow poorly^15,16^ and/or be unable to grow photoautotrophically^17,18^. Other neutral mutations in some C/G pairs in the target windows, which are the regions between the sequences that the left and right transcription activator-like effector (TALE) domains recognize, would also be expected.

Twelve ptpTALECD expression vectors were constructed (four pairs of CD halves for each of three targets). Each vector was introduced into *A. thaliana* and, 23 days after stratification (DAS), the targeted regions of the T_1_ plants were sequenced by Sanger sequencing. Only constructs from which T_1_ plants were obtained are shown in Fig. 1c–e and Supplementary Table 1a–c. In all three target windows, C/G pairs were replaced with T/A pairs in multiple T_1_ lines (Fig. 1c–h and Supplementary Table 1a–c). Surprisingly, in many lines, the targeted base(s) seemed to be homoplasmically substituted (homo), while in other lines, they seemed to be heteroplasmically or chimaerically substituted (h/c; Fig. 1c–h and Supplementary Table 1a–c). Such homoplasmic mutations might have occurred through stochastic sorting processes, such as selection of mutations in a small number of copies of plastid genomes (that is, plastid sorting)^19^ or gene conversion^20^. Nevertheless, it is also conceivable that ptpTALECD mutated the C/G pairs in the target windows of all plastid genomes at the early stage of embryogenesis because the *RPS5A* promoter used for ptpTALECD expression was reported to highly drive gene expression in egg cells and early embryos^21,22^. Not all C/G pairs in the target windows were substituted and the positions of the substituted C/G pairs were biased for all the three target windows (Fig. 1c–e). Three homoplasmically substituted bases were C of (5′)TC(3′) (Cp in Fig. 1c–e), which was the preferential target^1^ but a C of (5′)AC(3′) in *16S* *rRNA* gene was also substituted (Fig. 1c).

**Extended Data Fig. 3:**
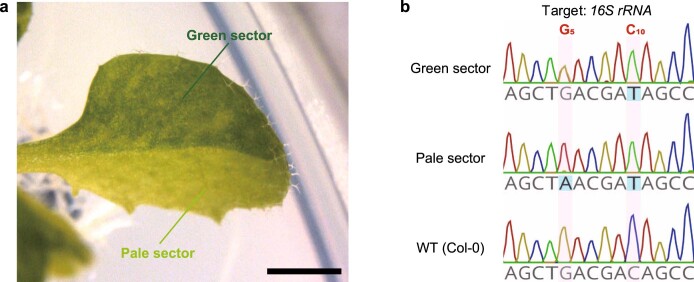
A chimerically base-edited leaf. A differently coloured leaf of 23 DAS *16S rRNA* 1397NC 3 (bar = 2 mm) plantlet (**a**) and its genotype at the ptpTALECD targeting window (**b**). The phenotype ‘half-pale variegation’ was observed in only one leaf on a T_1_ plant of the line *16S rRNA* 1397NC 3.

To investigate the stability of mutation rates during plant development, total DNAs extracted from an emerging leaf of T_1_ plants at 11 and 23 DAS (or from a cotyledon of slowly growing plants at 11 DAS; Supplementary Table 1a–c) were sequenced. Among the plants with base change(s) in the target window on either day, some had bases heteroplasmically or chimaerically (h/c) substituted on both days with their mutation rate increased or decreased (27.5%, 14/51; Fig. 1i) and others had bases at which the mutation rate differed between the two time points (for example, homoplasmy (homo) to h/c, 5.9% (3/51); h/c to null, 15.7% (8/51); h/c to homo, 11.8% (6/51); or null to h/c, 2.0% (1/51); Fig. 1i). Many of the remaining plants had bases that were homoplasmically substituted on both days (37.3%, 19/51; Fig. 1i). Interestingly, one leaf of one T_1_ plant (*16S* *rRNA* 1397NC 3) showed an example of sector formation^19^; that is, it had differently coloured sectors (wild-type-like green and pale) and the mutation rate at the Cp* in *16S* *rRNA* differed between the sectors (Extended Data Fig. 3a,b). Remarkably, most of the bases homoplasmically substituted at 11 DAS were also homoplasmically substituted at 23 DAS (86.4%, 19/22; Fig. 1i), suggesting that the targeted bases of T_1_ plants transformed by the ptpTALECD expression vector were homoplasmically substituted at a high frequency and that the homoplasmic mutations were stably fixed through development.

**Fig. 2:**
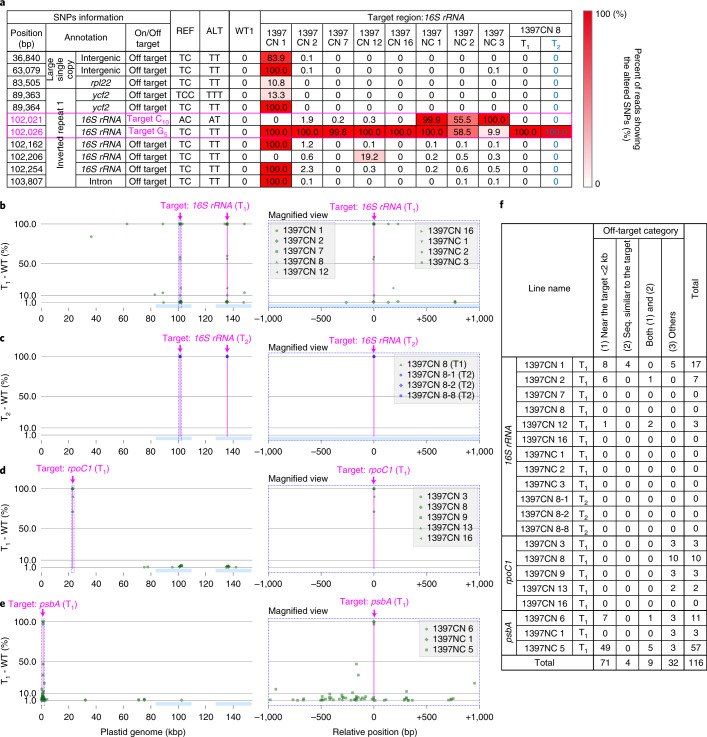
Investigation of on- and off-target mutations and determinants of off-target mutations. **a**, Mutated read frequencies in the plastid genomes of nine T_1_ lines (11 DAS) and one T_2_ line (49 DAS) targeted for *16S* *rRNA*. SNPs where ≥10% of the reads were different from the reference genome (AP000423.1) in at least one plant are listed. Supplementary Table 2 shows all the on- and off-target mutations in both organelle genomes, where different reads frequencies were ≥1%. **b**–**e**, Positions and frequencies of on- and off-target mutations (shown as green dots) in T_1_ plants targeting *16S* *rRNA* (**b**), *rpoC1* (**d**) and *psbA* (**e**) and in null-segregant T_2_ plants mutated in *16S* *rRNA* (**c**). Magenta lines represent the target windows. The right panels are magnified views of the regions surrounded by the dotted rectangles in left panels within 1,000 bp from the target nucleotides in each gene (G_5_ in *16S* *rRNA*, G_3_ in *rpoC1* and C_10_ in *psbA*). **f**, List of the off-target mutations categorized by types. Off-target mutations within 2 kb of the target windows and/or within 20 bp of sequences similar to those recognized by TALEs and in other regions are shown. In **b**–**f**, off-target mutations were defined as SNPs in which C/G-to-T/A substitutions (allele frequencies ≥0.01) were detected only in the T_1_ or T_2_ plants but not in three wild-type plants used as controls.

**Extended Data Fig. 4:**
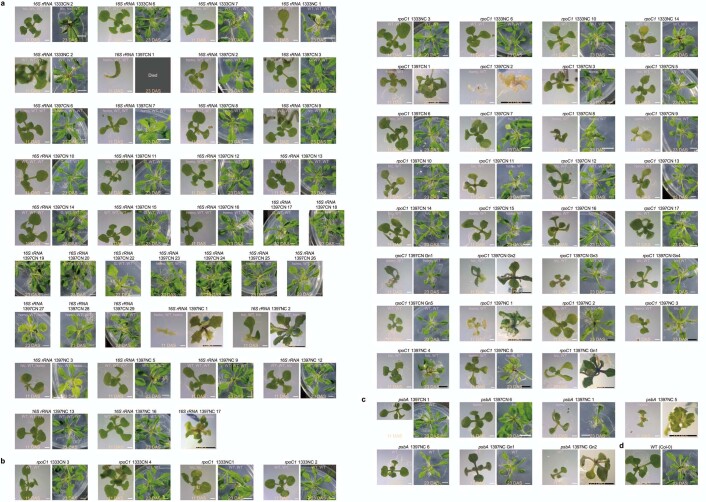
Appearance and genotypes of all T_1_ plants at 11 and 23 DAS in which C/Gs were substituted on at least one of the two days. The degrees of substitution at G_5_, G_8_, and C_10_ (*16S rRNA*, **a**), G_3_ and C_6_ (*rpoC1*, **b**), and C_10_ (*psbA*, **c**) are shown in magenta on the top of the images. All genotyped T_1_ plants are shown. **d**, Col-0 representative of ≥ four plants. Genotypes are shown in Supplementary Table 1. Bars = 1 mm (white bars on 11 DAS), and 5 mm (a black bar on 11 DAS and all bars on 23 DAS).

Next, we investigated the off-target effects of ptpTALECD on the plastid and mitochondrial genomes, because both organelle genomes are maternally inherited, so off-target mutations in these two organelle genomes cannot be segregated from the desired mutation by usual cross-breeding. The total genomes of 17 T_1_ plants were sequenced (Novaseq, illumina) and Fig. 2a and Supplementary Table 2 show the results. As Fig. 2a and Supplementary Table 2 show, the targeted C appeared to be homoplasmically substituted to T in 16 of them (*16S* *rRNA* 1397C-1397N (1397CN) 1, 2, 7, 8, 12, 16; 1397N-1397C (1397NC) 1~3; *psbA* 1397CN 6; 1397NC 1, 5; and *rpoC1* 1397CN 8, 9, 13, 16), while it was either heteroplasmically or chimaerically substituted in the remaining one (*rpoC1* 1397CN 3). Each redundant mutation in the inverted repeats of the plastid genome was counted as one mutation. The targeted bases in these 16 lines were confirmed to be homoplasmically or dominantly substituted and the base in the remaining one line was confirmed to be heteroplasmically or chimaerically substituted (Fig. 2a and Supplementary Table 2). Dominant off-target point mutations (with substitution frequencies >50%) were detected at six places in the line *16S* *rRNA* 1397CN 1, while no dominant off-target point mutations were detected in the other lines (Fig. 2a and Supplementary Table 2). The *16S* *rRNA* 1397CN 1 did not have any true leaves (Extended Data Fig. 4) and died before 23 DAS. A total of 116 off-target mutations (allele frequencies ≥1%) were detected in the plastid genomes (Fig. 2b–f and Supplementary Table 2). Most (69.0%) were located within 2,000 base pairs (bp) of the target windows while only a few (11.2%) were located within 20 bp of sequences similar to those recognized by TALEs. The rest were found in other regions. No dominant off-target mutations were detected in the mitochondrial genomes of these 17 lines, including *16S* *rRNA* 1397CN 1 (Supplementary Table 2). These results indicate that ptpTALECD only infrequently introduced off-target point mutations into organelle genomes and can specifically and homoplasmically (or dominantly) substitute C/G to T/A in the target windows.

**Fig. 3:**
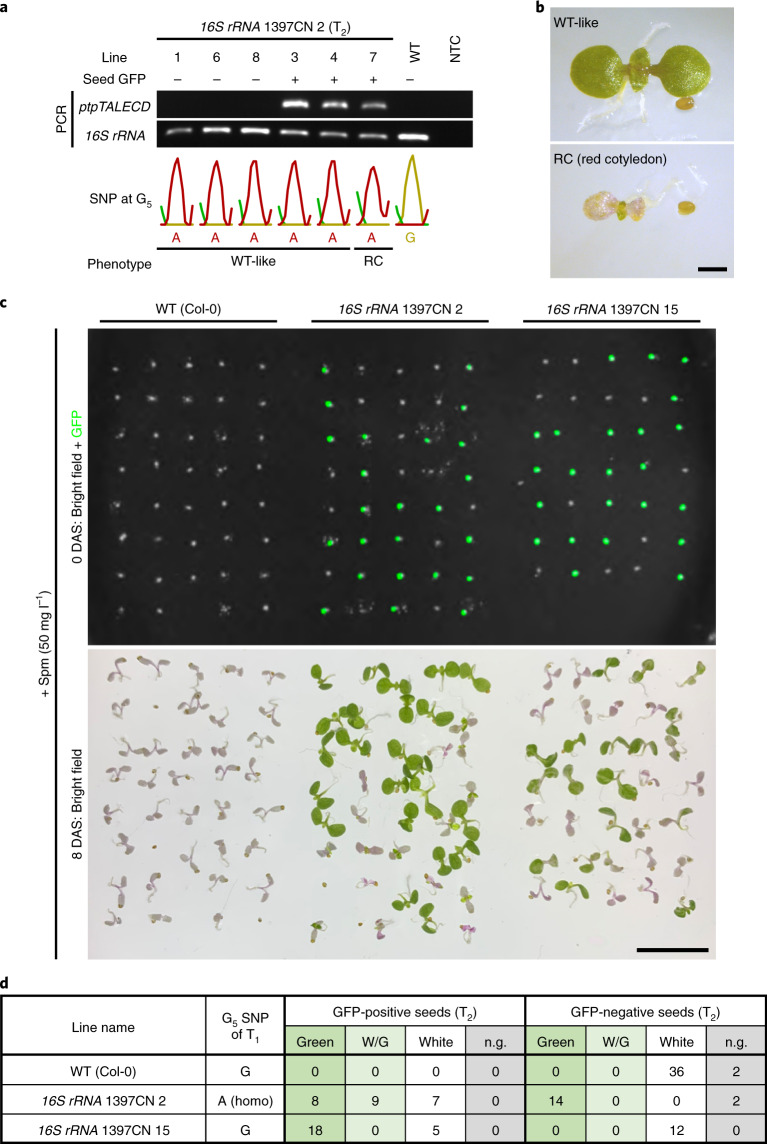
T_2_ generation analysis. **a**,**b**, Genotypes and phenotypes of T_2_ progenies of *16S* *rRNA* 1397CN 2. **a**, Gel images of bands of PCR products of *16S* *rRNA* and *ptpTALECD*, presence of seed GFP fluorescence, genotype**s** of G_5_ SNP and phenotypes of T_2_ progenies of *16S* *rRNA* 1397CN 2 are shown. Abbreviations: WT, wild type (Col-0); NTC, non-template control. **b**, Representative phenotype images of five WT-like plants (lines 1, 3, 4, 6, 8) and a plant with red cotyledons (line 7). Scale bar, 1 mm. **c**,**d**, Phenotypes of T_2_ progenies of *16S* *rRNA* 1397CN 2 and 15 in the presence of Spm. **c**, Images of seeds (0 DAS) and seedlings (8 DAS) of the T_2_ progenies of the two lines and WT (Col-0) on 1/2 MS medium containing 50 mg l^–1^ of Spm. Scale bar, 1 cm. **d**, A table that displays the relationship between the presence of seed GFP fluorescence and 8 DAS plants colour. Abbreviations: W/G, white or red cotyledons and green leaves; n.g., not germinated. Source data

**Extended Data Fig. 5:**
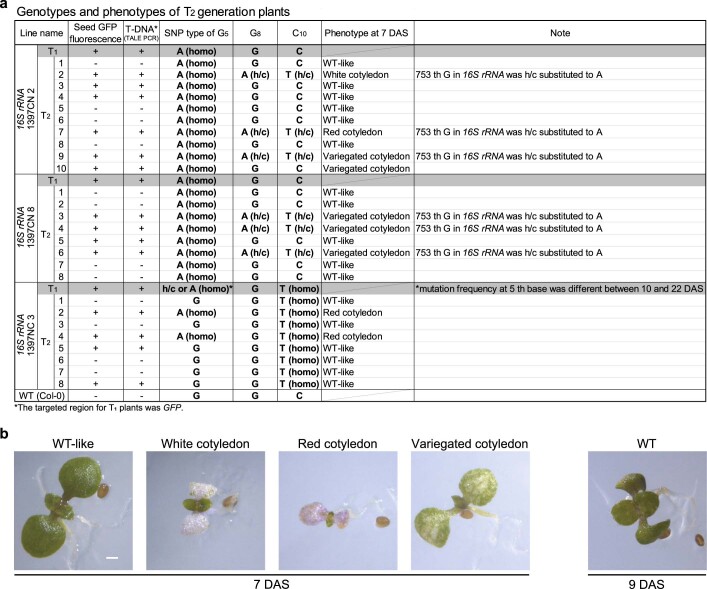
Genotypes and phenotypes of T_2_ progeny of T1 plants that had homoplasmic mutations in *16S rRNA*. **a**, Genotypes and phenotypes of T_2_ plants obtained from *16S rRNA* 1397CN 2, 8 and 1397NC 3 self-reproduction. **b**, Phenotype images of the T_2_ plants listed in (**a**). Representative images of at least three plants, except for the white cotyledon for which only one plant was available (bar = 0.5 mm).

**Extended Data Fig. 6:**
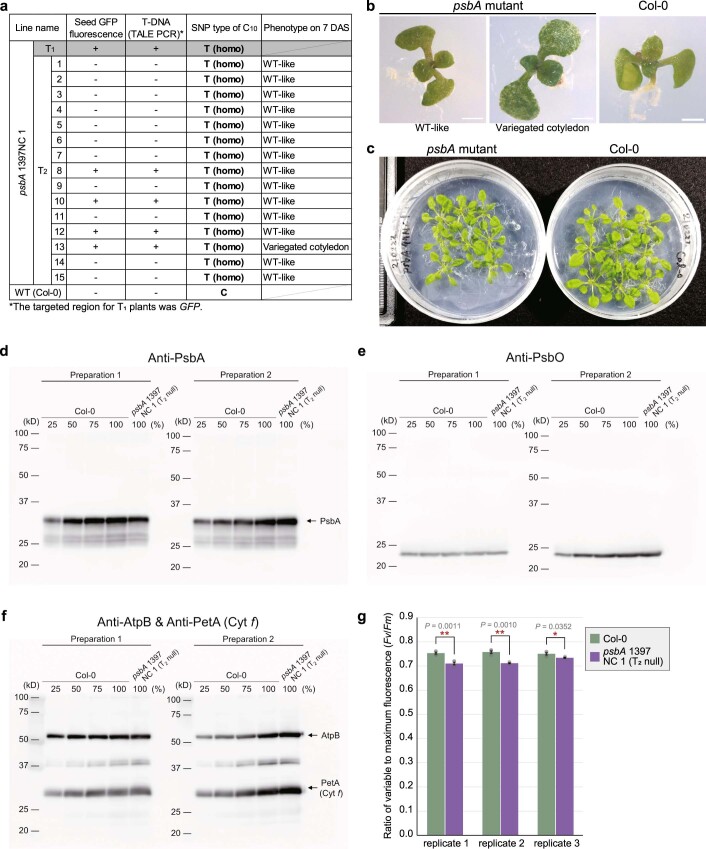
Genotypes and phenotypes of the progeny of *psbA* 1397NC 1. **a**, Genotypes of T_2_ progenies of *psbA* 1397NC 1. **b**,**c**, Representative phenotype images at 7 DAS of at least four plants except for variegated cotyledon for which only one plant was available (**b**) and 27 DAS (**c**). Bars = 1 mm in **b**, and diameter of plates = 9 cm in **c**. **d**-**f**, Immunoblot analysis of photosynthetic proteins in the thylakoid membrane of two independent experiments. Similar results were observed in each preparation. Labels on the lanes indicate the relative amounts of proteins loaded on the gel. 100 means proteins extracted from thylakoid membrane corresponding to 1 µg chlorophyll (**d**) or 2 µg of chlorophyll (**e** and **f**). **g**, Maximum quantum yield of PSII calculated as *Fv*/*Fm*. Chlorophyll fluorescence of four plants of each group (mutants and wild-type plants) were measured in each replicate. *P < 0.05 and **P < 0.01 (two-tailed Welch’s test). Error bars represent standard errors. Source data

**Extended Data Fig. 7:**
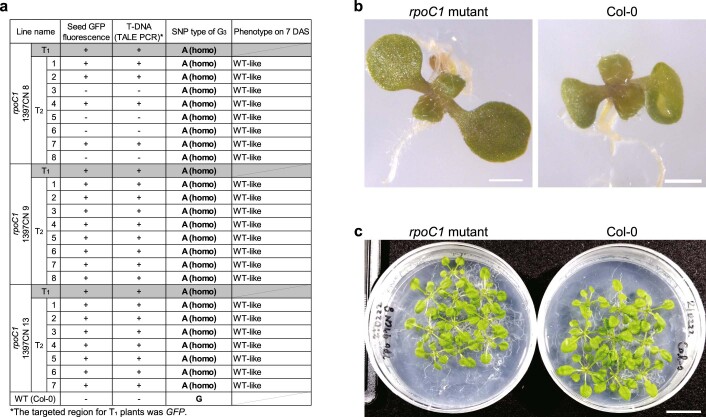
Genotypes and phenotypes of the progeny of *rpoC1* 1397CN 8, 9, and 13. **a**, Genotypes of T_2_ progenies of *rpoC1* 1397CN 8, 9, and 13. **b**,**c**, Representative phenotype images on 7 DAS (**b**, representative of ≥ four plants) and 27 DAS (**c**). Bars = 1 mm in **b**, and 2 cm in **c**.

All but one of the T_1_ plants that were transformed by the *16S* *rRNA*-targeting ptpTALECD vector and whose first Cp* (G_5_) and/or C_10_ was homoplasmically substituted were fertile (Supplementary Table 1a). The exception was *16S* *rRNA* 1397CN 1. To investigate whether the mutations were stably inherited by the offspring, the T_2_ progenies of three of these lines (*16S* *rRNA* 1397CN 2, 8 and 1397NC 3) were genotyped (Fig. 3a and Extended Data Fig. 5a). Transgenic T_2_ plants were identified by having seed-specific green fluorescent protein (GFP) fluorescence from *Ole1 pro*::*Ole1*-*GFP* (ref. ^23^) on the transfer DNA (T-DNA; Fig. 1b) and/or a positive polymerase chain reaction (PCR) result showing the presence of the *ptpTALECD* reading frame (Fig. 1b and 3a). Both progeny stably inherited the homoplasmic mutations (Fig. 3a and Extended Data Fig. 5a). Interestingly, some T_2_ plants had white, red or variegated cotyledons (Fig. 3b and Extended Data Fig. 5b), which were different from the phenotypes of their parents (Extended Data Fig. 4). All of these plants were GFP positive (Fig. 3a and Extended Data Fig. 5a) and many of them (8/9) had additional mutation(s) in or near the target window of *16S* *rRNA* (Extended Data Fig. 5a). Because, as mentioned above, the *RPS5A promoter* used for ptpTALECD expression was reported to highly drive gene expression in egg cells and early embryos^21,22^, de novo mutagenesis may have occurred during the early developmental stage in these transgenic T_2_ plants with abnormal cotyledons. In contrast, all T_2_ plants of the T-DNA-free null segregants examined had the targeted mutations without any of the additional altered phenotypes described above. No major off-target mutations were detected in the three null-segregant T_2_ plants (*16S* *rRNA* 1397CN 8 lines 1, 2 and 8), whose genomes were sequenced by next generation sequencing (Fig. 2a and Supplementary Table 2). Homoplasmic mutations in *rpoC1* (G_3_) and *psbA* (C_10_) in other lines were also inherited by their T_2_ progeny (Extended Data Figs. 6a and 7a). These data indicate that plastid genomes with artificially introduced point mutations were stably inherited, independently of nuclear T-DNA inheritance and also suggest that transgene-free plants with targeted point mutations in the plastid genomes were successfully established.

**Extended Data Fig. 8:**
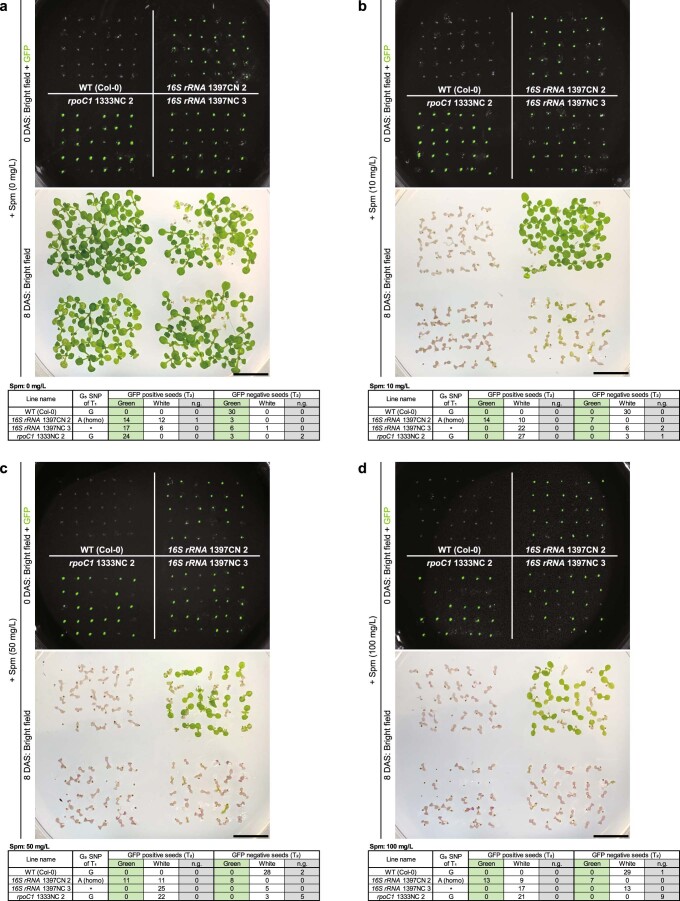
Spectinomycin (Spm) resistance of T_2_ plants. **a**-**d**, Seeds and 8 DAS seedlings images of Col-0 (upper left) and T_2_ progenies of *16S rRNA* 1397CN 2 (upper right), 1397NC 3 (lower right), and *rpoC1* 1333NC 2 (lower left) on 1/2 MS medium containing 0 (**a**), 10 (**b**), 50 (**c**), or 100 (**d**) mg/L Spm are shown. We show merged images of a seeds image and a seeds GFP fluorescence image. Bars = 1 cm. Tables show the relationship between seed GFP and cotyledons colour on 8 DAS. n.g.: not germinated. *means that T_1_ G_5_ SNP was different between 11 and 23 DAS (Supplementary Table 1a).

**Extended Data Fig. 9:**
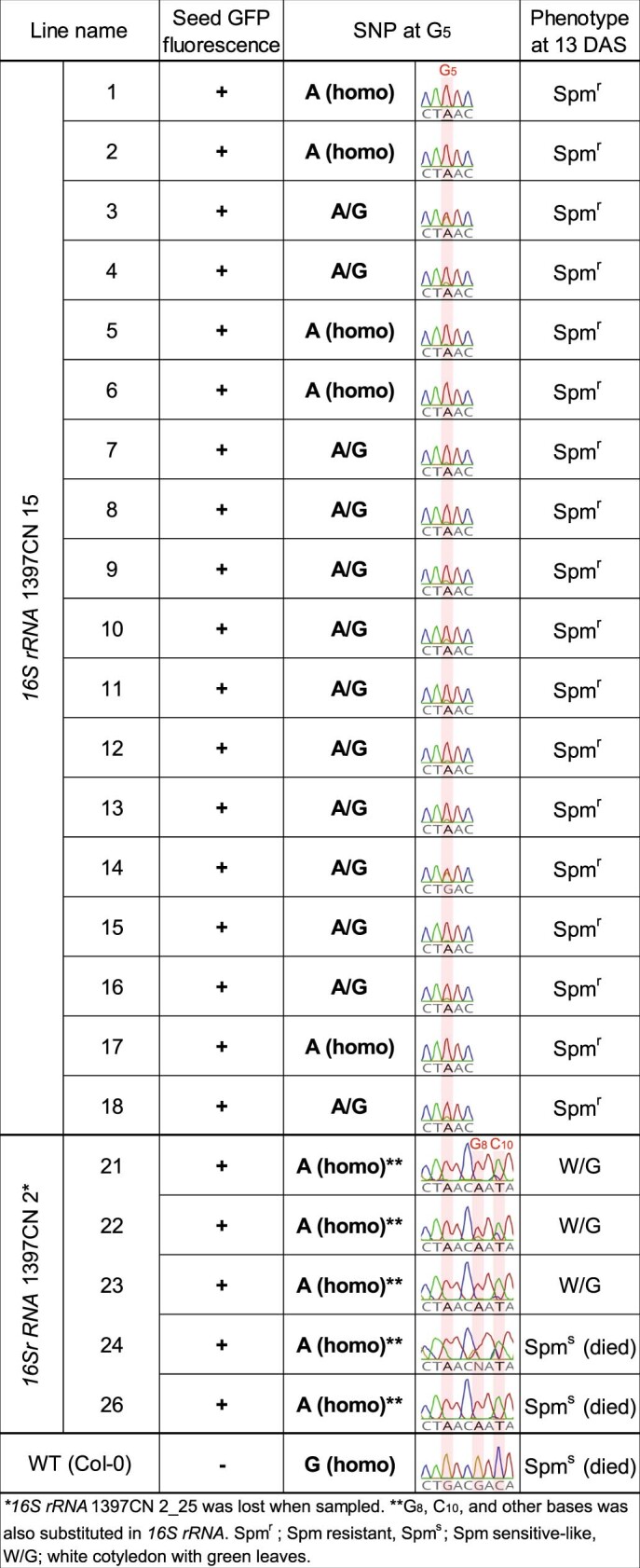
Cotyledon genotypes of 13 DAS Spm^r^ and Spm^s^-like plants. The presence of seed GFP, SNP at G_5_, and phenotypes of 13 DAS Spm^r^ plants (T_2_ of *16S rRNA* 1397CN 15) and Spm^s^-like plants (T_2_ of *16S rRNA* 1397CN 2) in Fig. 3c are shown.

The antibiotic Spm binds to a specific location in *Escherichia coli 16S* *rRNA* and inhibits translation^24^. Substitution of a specific G near this region to A confers Spm resistance (Spm^r^)^13^. The targeted G_5_ in the *Arabidopsis* plastid *16S* *rRNA* gene is homologous to this G. Several mutations are known to confer Spm^r^ to flowering plants^25,26^ but none of them occur at the position of the targeted G_5_. T_2_ seeds obtained from a T_1_ plant in which G_5_ was homoplasmically substituted to A (*16S* *rRNA* 1397CN 2; Supplementary Table 1a) were sown on plates containing Spm. Many of the seedlings that germinated from these seeds showed Spm^r^, regardless of the presence of seed GFP fluorescence (Fig. 3c and Extended Data Fig. 8b–d). However, some T_2_ progenies from *16S* *rRNA* 1397CN 2 showed a Spm-sensitive (Spm^s^)-like phenotype (white plantlet with purple cotyledon; Fig. 3c and Extended Data Fig. 8a–d). All the Spm^s^-like plantlets germinated from GFP-positive seeds (Fig. 3c and Extended Data Fig. 8a–d) and many of them (5/5; Extended Data Fig. 9) had multiple de novo mutations (in addition to G_5_) in *16S* *rRNA*. This suggests that the de novo mutations caused dysfunction of *16S* *rRNA*, resulting in the Spm^s^-like phenotype. The existence of Spm^s^-like T_2_ plants of *16S* *rRNA* 1397CN 2 on Spm-free medium (Extended Data Fig. 5a,b) with the de novo mutation(s) support this suggestion. Surprisingly, some of the progeny of a T_1_ plant (*16S* *rRNA* 1397CN 15) that had the G_5_ mutation at very low frequency at 11 DAS and no mutation at 23 DAS (Supplementary Table 1a) also showed Spm^r^. These progeny germinated from GFP-positive seeds (Fig. 3c). In five of them, the G_5_ was homoplasmically substituted to A and in 13 others it was dominantly substituted to A (Extended Data Fig. 9). This suggests that the inherited nuclear T-DNA caused a major de novo mutation on the G_5_. These results suggest that homoplasmic substitution of G_5_ to A confers Spm^r^ to *A. thaliana*. Furthermore, the result that the GFP-negative T_2_ progeny showed Spm^r^ or Spm^s^ phenotype that was predictable from SNPs at G_5_ in the T_1_ plants showed that the null-segregant T_2_ plants were likely to inherit mutation(s) that their parent had and not likely to have additional mutations.

Previous studies showed that accumulation of D1 protein (encoded by *psbA*) and/or the maximum quantum yield of PSII (*Fv/Fm*) drastically decreased in mutants deficient in *psbA* expression^17,18^. Furthermore, these mutants looked pale^17^ and could not grow photoautotrophically^17,18^. Surprisingly, *psbA* 1397NC 1, which had the homoplasmic mutation at the *psbA* initiation codon (C_10_) at both 11 and 23 DAS (Supplementary Table 1c), could grow photoautotrophically and set viable seeds. Thus, to investigate the effects of the homoplasmic mutation at the *psbA* initiation codon (C_10_) on its expression, we measured *Fv/Fm* and the accumulation of D1 protein in T-DNA-free null-segregant T_2_ progeny of *psbA* 1397NC 1, which were confirmed to inherit the homoplasmic mutation. Unexpectedly, their growth (Extended Data Fig. 6b,c) and accumulation of D1 protein (Extended Data Fig. 6d–f) were comparable to those in wild-type plants, while *Fv/Fm* was only slightly decreased compared with wild-type plants (Extended Data Fig. 6g). One possibility is that another codon served as the initiation codon. It could be another AUG, or possibly a GUG or UUG, which can also serve as start codons in the chloroplast^27^. Upstream of the altered AUA, no such sites occur after the nearest stop codon. Downstream, the next potential start codons would shorten the protein by at least 10% but they can be excluded because the recombinant protein was the same size as the wild-type protein (Extended Data Fig. 6d). These results suggest that the AUA codon does not greatly affect the initiation of translation of *psbA* or the D1 level but that the AUG codon is necessary for the full activity of PSII. Thus, a better way to knock out a plastid gene might be to create a premature stop codon in its reading frame rather than to change the initiation codon to AUA. In *rpoC1*, none of the homoplasmic mutations that were obtained were at the initiation codon as expected. Instead, they were at the second codon where they caused a synonymous mutation (Ile to Ile; Fig. 1d and Supplementary Table 1b). Null-segregant T_2_ progeny of *rpoC1* 1397CN 8, which had the synonymous homoplasmic mutation at both 11 and 23 DAS, inherited the homoplasmic mutation and appeared to grow as well as wild-type plants (Extended Data Fig. 7b,c).

These experiments showed that ptpTALECD could specifically introduce homoplasmic C-to-T mutations in target windows in the *A. thaliana* plastid genome and that the mutations were stably (and probably maternally) inherited by the progeny seeds. Previous attempts to introduce homoplasmic mutations in mammalian mitochondrial genomes were unsuccessful^1,28^. The method was also successful in a region of inverted repeats, where mutations are thought to occur at a lower rate due to their greater potential for copy correction^29^; *16S* *rRNA* occurs in inverted repeats and targeted point mutations were successfully introduced in both copies. Compared to traditional methods for plastid transformation, such as biolistic methods, ptpTALECD technology has three advantages. First, it allows plastid-genome editing of *A. thaliana* without using specific mutants^3,4^ or a specific ecotype^30^ and without tissue culture, which is a major obstacle to plastid transformation. Second, it could probably be used to edit plastid genomes of other plant species that are recalcitrant to plastid transformation but amenable to nuclear transformation. And third, it could be used to create plastid-genome-edited plants without leaving any foreign gene in their genomes. Such plants are not regarded as GMOs in several countries. On the other hand, the ptpTALECD method has some problems with respect to accuracy. For example, unwanted substitutions in the target windows occurred (C_10_ in *16S* *rRNA* and G_3_ in *rpoC1*; Fig. 1c,d), while homoplasmic mutations at some special target C/G pairs in the target windows were not introduced (G_8_ in *16S* *rRNA* and C_6_ in *rpoC1*; Fig. 1c,d). These problems might be avoided by sliding the TALE recognition targets a few base pairs upstream or downstream or by using different sizes of target windows or by optimizing the sequences linking the TALE and CD^31^. In any case, only a few mutations in this study were off target. We also obtained null-segregant T_2_ plants that had the targeted homoplasmic mutation but had no off-target mutations (Fig. 2a and Supplementary Table 2).

This technology may also be useful for strengthening agronomic traits. For example, amino acid polymorphisms in the plastid-encoded ribulose 1,5-bisphosphate carboxylase/oxygenase (Rubisco) large subunit are expected to affect the carbon assimilation (and oxidation) rate^32,33^ and some polymorphisms in *psbA* (not involving the C_10_ in this study) enhance herbicide resistance^34^. In addition, null-segregant plants are not regarded as GMOs in some countries and the introduced mutations would not leak out of the pollen^2,5^. Therefore, plants with their plastid genomes precisely edited by ptpTALECD might be more acceptable to the public. Also, this technology could be used for creating premature stop codons, substituting amino acids and modifying RNA editing sites. Thus, ptpTALECD technology has the potential to accelerate both plant breeding and basic research on plastid-encoded genes.

## Methods

### Plant material and growth conditions

*A. thaliana* Columbia-0 (Col-0) and transgenic plants were grown at 22°C and under long-day conditions (16 h light, 8 h dark). Col-0 seeds were sown on 1/2 Murashige and Skoog (MS) medium (pH 5.7) containing 2.3 g l^−1^ of MS Plant Salt Mixture (Wako), 500 mg l^−1^ of MES, 10 g l^−1^ of sucrose, 1 ml l^−1^ of Plant Preservative Mixture (Plant Cell Technology), 1 ml l^−1^ of Gamborg’s Vitamin Solution (Sigma–Aldrich) and 8 g l^−1^ of agar. Seedlings at 2–3-weeks-old were transferred to Jiffy-7 (Jiffy Products International) and thereafter subjected to *Agrobacterium* transfection. Most T_1_ plants were transplanted to Jiffy-7 but several growth**-**retarded plants were transplanted to plant boxes containing the 1/2 MS medium at 23 DAS.

### Designing the TALE binding sequence

The TALE targeting sequence was designed to be on both sides of the CD targeting window, with Old TALEN Targeter (https://tale-nt.cac.cornell.edu/node/add/talen-old). The first recognized base was required to be adjacent to the 3′ side of ‘T’ as far as possible. The minimum length of TALE targeting sequence was 15 bp so that TALE would specifically bind the sequence. All the sequences that TALE binds and the target windows between the TALE binding sequences are shown in Supplementary Table 3 and Fig. 1c–e.

### Vector constructions

A pair of left and right ptpTALECDs in Ti-plasmids (Extended Data Fig. 1b) for each target was constructed by using Platinum Gate assembling kit and multisite Gateway (Thermo Fisher) as described in our previous study of mitochondria-targeted TALEN^11^.

**Extended Data Fig. 10:**
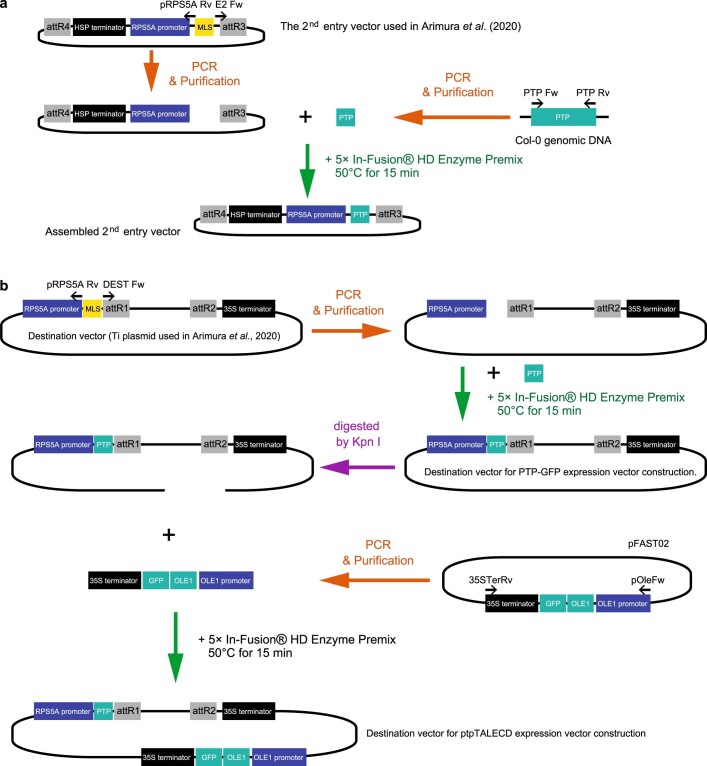
The ^2nd^ entry vector and destination vector construction. **a**, The 2^nd^ entry vector construction. The 2^nd^ entry vector used in Arimura et al.^10^, and RECA1 plastid transit peptide coding sequence (PTP; Supplementary Table 4) were amplified with primers corresponding to the DNA template (Supplementary Table 5). The purified PCR products (1: PTP + 2^nd^ entry vector, 2: PTP + destination vector) were mixed with 5× In-Fusion HD Cloning Enzyme Premix (TaKaRa) and incubated at 50 °C for 15 minutes. **b**, The destination vector construction. The destination vector used in Arimura *et al*. (2020) was amplified with primers (Supplementary Table 5); purified PCR product was mixed with the PTP PCR product and 5× In-Fusion HD Cloning Enzyme Premix and incubated at 50 °C for 15 minutes to make the destination vector for PTP-GFP expression vector construction. The assembled destination vector was digested by KpnI and the purified product was incubated at 50 °C for 15 minutes after mixed with 5× In-Fusion HD Cloning Enzyme Premix and OLE1-GFP coding sequence amplified from pFAST02 (INPLANTA INNOVATIONS INC), to make the destination vector for ptpTALECD expression vector construction.

The DNA binding domains of ptpTALECD were assembled with the Platinum Gate TALEN system^6^ on the basis of the same previous study^11^ (Extended Data Fig. 1a). Each FokI coding sequence in the previous vectors of mitoTALENs used for assembly-step2 was replaced in advance by the CD half and UGI coding sequence with In-Fusion HD Cloning Kit (TaKaRa; Extended Data Fig. 2). The CD half and UGI coding sequences were designed to encode the same amino acids as those of Mok’s experiment^1^ and artificially synthesized by Eurofins Genomics with the codon usage optimized for *A. thaliana* (https://www.eurofinsgenomics.jp/jp/orderpages/gsy/gene-synthesis-multiple/; Supplementary Table 4). The reading frames in the assembled first and third entry vectors and the second entry vector (below) were transferred into the Ti plasmid^10^ by a multi-LR reaction with LR Clonase II Plus enzyme (Thermo Fisher Scientific; Extended Data Fig. 1b). The second entry vector had an *Arabidopsis* heat-shock protein terminator^35^, an *Arabidopsis RPS5A* promoter and the N terminal (51 aa) PTP of *Arabidopsis RECA1* (refs. ^7,8^; Extended Data Fig. 10a). This Ti plasmid was made from a Gateway destination Ti plasmid pK7WG2 (ref. ^36^) by replacing the CaMV 35S promoter with the *Arabidopsis RPS5A* promoter and inserting the PTP coding sequence and *Ole1* pro::*Ole1*-*GFP* derived from pFAST02 (ref. ^23^; Extended Data Fig. 10b; http://www.inplanta.jp/pfast.html). All primers used for vector construction are listed in Supplementary Table 5. All plasmids are deposited in Addgene and their sequences are also available in Addgene (ID 171723–171736).

### Plant transformation and screening transformants

Col-0 plants were transformed by floral dipping^12^ with *Agrobacterium tumefaciens* strain C58C1 that harboured one of the transformation vectors described above. Transgenic T_1_ seeds were selected at first by observing seed GFP fluorescence. GFP-positive seeds were sown on the 1/2 MS medium (section Plant material and growth conditions) further containing 125 mg l^−1^ of claforan. In addition, GFP-negative seeds were sown on the 1/2 MS medium containing 50 mg l^−1^ of kanamycin and 125 mg l^−1^ of claforan.

### Sanger sequencing and next generation sequencing and their analyses

Total DNAs were extracted from an emerging true leaf or a cotyledon of the selected seedlings with Maxwell RSC Plant DNA Kit (Promega). To genotype transgenic lines, plastid DNA sequences adjacent to the CD targeting windows were amplified with primer sets (Supplementary Table 6). Purified PCR products were subjected to Sanger sequencing (Eurofins Genomics) to detect substitution of the targeted bases. The data were analysed with Geneious prime (v.2020.2.2).

We called SNPs in the plastid and mitochondrial genomes using total DNA sequenced data. First, we ordered Macrogen Japan to prepare paired-end libraries using a Nextera XT DNA library Prep Kit (Illumina) and sequenced using Illumina NovaSeq 6000 platform. As preprocess for analysis, low-quality and adaptor sequences in the reads were trimmed using Platanus_trim v.1.0.7 (http://platanus.bio.titech.ac.jp/pltanus_trim). Pair-end reads of each strain were mapped to reference sequences (AP000423.1 and BK010421.1) using BWA (v.0.7.12)^37^ in single-ended mode. We filtered out inadequate mapped reads with mapping identities ≤97% or alignment cover rates ≤80%. SNPs were then called using samtools mpileup command (-uf -d 30000 -L 2000) and bcftools call command (-m -A -P 0.1)^38^. We finally listed positions in which variants with allele frequencies (AFs) ≥0.1 were detected in at least one strain including the WT (Fig. 2a). SNP calls with AFs ≥0.01 were also performed for positions with read depths ≥500 (Supplementary Table 2).

To evaluate whether closeness to target sites or similarity to TALE sequences influenced the locations of off-target mutations, we tallied off-target mutations that were either within 2,000 bp of the target site or within 20 bp of sequences ≥70% similar to those recognized by one of the TALEs.

### Genotyping T_2_ plants

T_2_ seeds gained from several T_1_ lines were sown on the 1/2 MS medium (section Plant material and growth conditions). The genotypes of the target windows of a cotyledon of the 7 DAS (for Fig. 3a and Extended Data Figs. 5a, 6a and 7a) or 13 DAS (for Extended Data Fig. 9) seedlings were determined in the same way as determining those of T_1_ plants (above). The *ptpTALECD* PCRs were performed with primers described in Supplementary Table 6.

### Screening Spm-resistant plants

T_2_ seeds obtained from a T_1_ line of which G_5_ in *16S* *rRNA* was homoplasmically substituted at 11 and 23 DAS and control seeds were sown on the 1/2 MS medium (section Plant material and growth conditions) containing 0, 10, 50 or 100 mg l^−1^ of Spm (without Plant Preservative Mixture for Extended Data Fig. 8a–d). Phenotypes of germinated seedlings were observed on 8 DAS.

### Measurement of chlorophyll fluorescence

Chlorophyll fluorescence was measured using a MINI-pulse-amplitude modulation portable chlorophyll fluorometer (MINI-PAM; Walz). Minimal fluorescence at open PSII centres in the dark-adapted state (*F*_o_) was excited by a weak measuring light (650 nm) at a photon flux density of 0.05 to 0.1 μmol of photons m^−2^ s^−1^. A saturating pulse of white light (800 ms, 8,000 μmol of photons m^−2^ s^−1^) was applied to determine the maximal fluorescence at closed PSII centres in the dark-adapted state (*F*_m_). Maximum quantum yield of PSII was calculated as *F*_v_/*F*_m_. These procedures were done independently three times (experimental replicates = 3). In each replicate, four plants of each genotype (Col-0 and *psbA* 1397NC 1 T_2_) were analysed, average values and standard errors were calculated and *F*_v_/*F*_m_ values of the two groups were tested by two-tailed Welch’s test.

### SDS–polyacrylamide gel electrophoresis and immunoblot analyses

Leaf extract was prepared by grinding the rosette leaves using mortar and pestle in an ice-cold buffer (20 mM Tricine (pH 8.4) containing 330 mM sorbitol, 10 mM NaHCO_3_, 5 mM EGTA and 5 mM EDTA). After filtration with two layers of Miracloth, intact chloroplasts were collected by centrifugation for 5 min at 4,800*g*. The purified chloroplasts were ruptured in a buffer (20 mM HEPES-KOH (pH 7.6), 5 mM MgCl_2_, 2.5 mM EDTA and complete ULTRA protease-inhibitor cocktail (Roche)). The insoluble fraction containing thylakoids and envelopes was separated from the soluble fraction by centrifugation for 2 min at 15,000*g* and resuspended in the above buffer. The concentration of chlorophyll was determined as described previously^39^. Chloroplast thylakoid and membrane proteins were solubilized in SDS–PAGE sample buffer. Proteins solubilized from the thylakoid membrane corresponding to 1–2 μg of chlorophyll were separated by 12.5% (w/v) SDS–PAGE and electrotransferred onto polyvinylidene fluoride membranes. The antibodies were added and the protein–antibody complexes were labelled using the ECL Prime western-blotting detection system (GE Healthcare). The chemiluminescence was detected with a lumino-image analyser (LAS4000, GE Healthcare). Anti-PsbA and anti-AtpB were purchased from Agrisera. Anti-PetA and anti-PsbO were kindly provided by A. Makino (Tohoku University, Japan) and T. Endo (Kyoto University, Japan), respectively.

### Image processing

Plant images were taken by iPhone Xs (Apple) and LEICA MC 170 HD (Leica). Gel images were taken by ChemiDoc MP Imaging System (BIORAD). These images were processed with Adobe Photoshop 2021 (Adobe). Figures and tables were made with Adobe Photoshop 2021 and Adobe Illustrator 2021 (Adobe).

### Reporting Summary

Further information on research design is available in the Nature Research Reporting Summary linked to this article.

## Supplementary information

Reporting Summary

Supplementary Tables Supplementary Tables 1–6.

## Data Availability

All data are available in the main text or the Supplementary Information. Source data are provided with this paper.

## Source data

Source Data Fig. 3 (1) Unprocessed gel image (*TALECD*). (2) Unprocessed gel image (16S rRNA). Source Data Extended Data Fig. 6g Statistical data.
