# Supplementary material for: Targeted base editing in the plastid genome of Arabidopsis thaliana
Source: Nat Plants. 2021 Jul 1;7(7):906–13. doi: 10.1038/s41477-021-00954-6 (PMC8289735; doi:10.1038/s41477-021-00954-6)
Supplement: Supplementary file 1 — Reporting Summary [file 41477_2021_954_MOESM1_ESM.pdf]

nature research

Corresponding author(s): Shin-ichi Aimura

Last updated by author(s): 2021/5/20

Reporting Summary

Nature Research wishes to improve the reproducibility of the work that we publish. This form provides structure for consistency and transparency in reporting. For further information on Nature Research policies, see our Editorial Policies and the Editorial Policy Checklist.

Statistics

For all statistical analyses, confirm that the following items are present in the figure legend, table legend, main text, or Methods section.

n/a	Confirmed	
☐	☒	The exact sample size (n) for each experimental group/condition, given as a discrete number and unit of measurement
☐	☒	A statement on whether measurements were taken from distinct samples or whether the same sample was measured repeatedly
☐	☒	The statistical test(s) used AND whether they are one- or two-sided<br>*Only common tests should be described solely by name; describe more complex techniques in the Methods section.*
☒	☐	A description of all covariates tested
☒	☐	A description of any assumptions or corrections, such as tests of normality and adjustment for multiple comparisons
☒	☐	A full description of the statistical parameters including central tendency (e.g. means) or other basic estimates (e.g. regression coefficient) AND variation (e.g. standard deviation) or associated estimates of uncertainty (e.g. confidence intervals)
☐	☒	For null hypothesis testing, the test statistic (e.g. F, t, r) with confidence intervals, effect sizes, degrees of freedom and P value noted<br>*Give P values as exact values whenever suitable.*
☒	☐	For Bayesian analysis, information on the choice of priors and Markov chain Monte Carlo settings
☒	☐	For hierarchical and complex designs, identification of the appropriate level for tests and full reporting of outcomes
☒	☐	Estimates of effect sizes (e.g. Cohen's d, Pearson's r), indicating how they were calculated

Our web collection on statistics for biologists contains articles on many of the points above.

Software and code

Policy information about availability of computer code

Data collection	Sanger sequencings were performed by Eurofins Genomics. Whole genome sequencing was performed by Macrogen Japan using an Illumina Novaseq platform. No software was used for data collection.
Data analysis	Platanus_trim v1.0.7, Geneious prime (v 2020.2.2), and BWA (v 0.7.12) were used to analyze sequencing data. Adobe Photoshop 2021 and Adobe Illustrator 2021 were used to prepare figures and tables.

For manuscripts utilizing custom algorithms or software that are central to the research but not yet described in published literature, software must be made available to editors and reviewers. We strongly encourage code deposition in a community repository (e.g. GitHub). See the Nature Research guidelines for submitting code & software for further information.

Data

Policy information about availability of data

All manuscripts must include a data availability statement. This statement should provide the following information, where applicable:

- Accession codes, unique identifiers, or web links for publicly available datasets
- A list of figures that have associated raw data
- A description of any restrictions on data availability

All data supporting the findings of this study are available in the article and the supplementary information. Vector sequences will be deposited in GenBank and addgene. Accession numbers of the reference sequences for organelle genome used in this study are AP000423.1 (plastid) and BK010421.1 (mitochondrion).

Field-specific reporting

Please select the one below that is the best fit for your research. If you are not sure, read the appropriate sections before making your selection.

☒ Life sciences ☐ Behavioural & social sciences ☐ Ecological, evolutionary & environmental sciences

For a reference copy of the document with all sections, see nature.com/documents/nr-reporting-summary-flat.pdf

Life sciences study design

All studies must disclose on these points even when the disclosure is negative.

Sample size	At least seven T1 plants for each targeted gene were analyzed. At least eight T2 progeny of three T1 lines were genotyped. To investigate spectinomycin resistance, at least 30 seeds per line were sown on one plate. The number of T1 plants used was determined by the maximum number that could be generated in our growth cabinets. The numbers of T2 seeds used was determined by the the maximum number of seeds (T2) available at the time of the experiment.
Data exclusions	No data exclusion.
Replication	For each targeted gene, at least seven T1 lines were genotyped and targeted bases were successfully substituted in at least three lines. Total DNA NGS was successfully performed on 17 T1 lines, 3 T2 plants and 3 wild-type plants. At least eight T2 progeny of the seven T1 lines were genotyped and all data are shown. Spectinomycin resistance was assayed four times, with the same results. Chlorophyll fluorescence measurements and Immunoblot experiments were successfully done in triplicate and duplicated respectively.
Randomization	All T1 plants were subjected to Sanger sequencing. The samples that were subjected to total DNA NGS were chosen randomly among the plants that appeared to have a homoplasmic mutation at the targeted base based on Sanger sequencing. T2 seeds used for genotyping were selected randomly among those whose T1 parents had a homoplasmic mutation in the target window. T2 seeds used for spectinomycin resistance assay were selected randomly among those whose T1 parents had a homoplasmic mutation at G5 in 16S rRNA. T2 progeny of psbA 1397NC 1 that were subjected to chlorophyll measurements and immunoblot experiments were selected randomly among the null segregants of T-DNA.
Blinding	Blinding was not required because all analyses including genotyping by sequencing and phenotyping by antibiotics and molecular methods could be carried out without making any subjective judgements.

Reporting for specific materials, systems and methods

We require information from authors about some types of materials, experimental systems and methods used in many studies. Here, indicate whether each material, system or method listed is relevant to your study. If you are not sure if a list item applies to your research, read the appropriate section before selecting a response.

Materials & experimental systems

n/a	Involved in the study	
☐	☒	Antibodies
☒	☐	Eukaryotic cell lines
☒	☐	Palaeontology and archaeology
☒	☐	Animals and other organisms
☒	☐	Human research participants
☒	☐	Clinical data
☒	☐	Dual use research of concern

Methods

n/a	Involved in the study	
☒	☐	ChIP-seq
☒	☐	Flow cytometry
☒	☐	MRI-based neuroimaging

Antibodies

Antibodies used	PsbA (AS11 1786, Agrisera); AtpB (AS05 085, Agrisera); PsbO (Murakami et al., FEBS Lett., 523: 138-142, 2002); Cyt f (Hidema et al., Plant Physiol., 97: 1287-1293, 1991).
Validation	Antibodies against PsbA, AtpB, PsbO or Cyt f can detect Arabidopsis proteins.
