# Supplementary figures and images for: Targeted base editing in the plastid genome of Arabidopsis thaliana
Source: Nat Plants. 2021 Jul 1;7(7):906–13. doi: 10.1038/s41477-021-00954-6 (PMC8289735; doi:10.1038/s41477-021-00954-6)

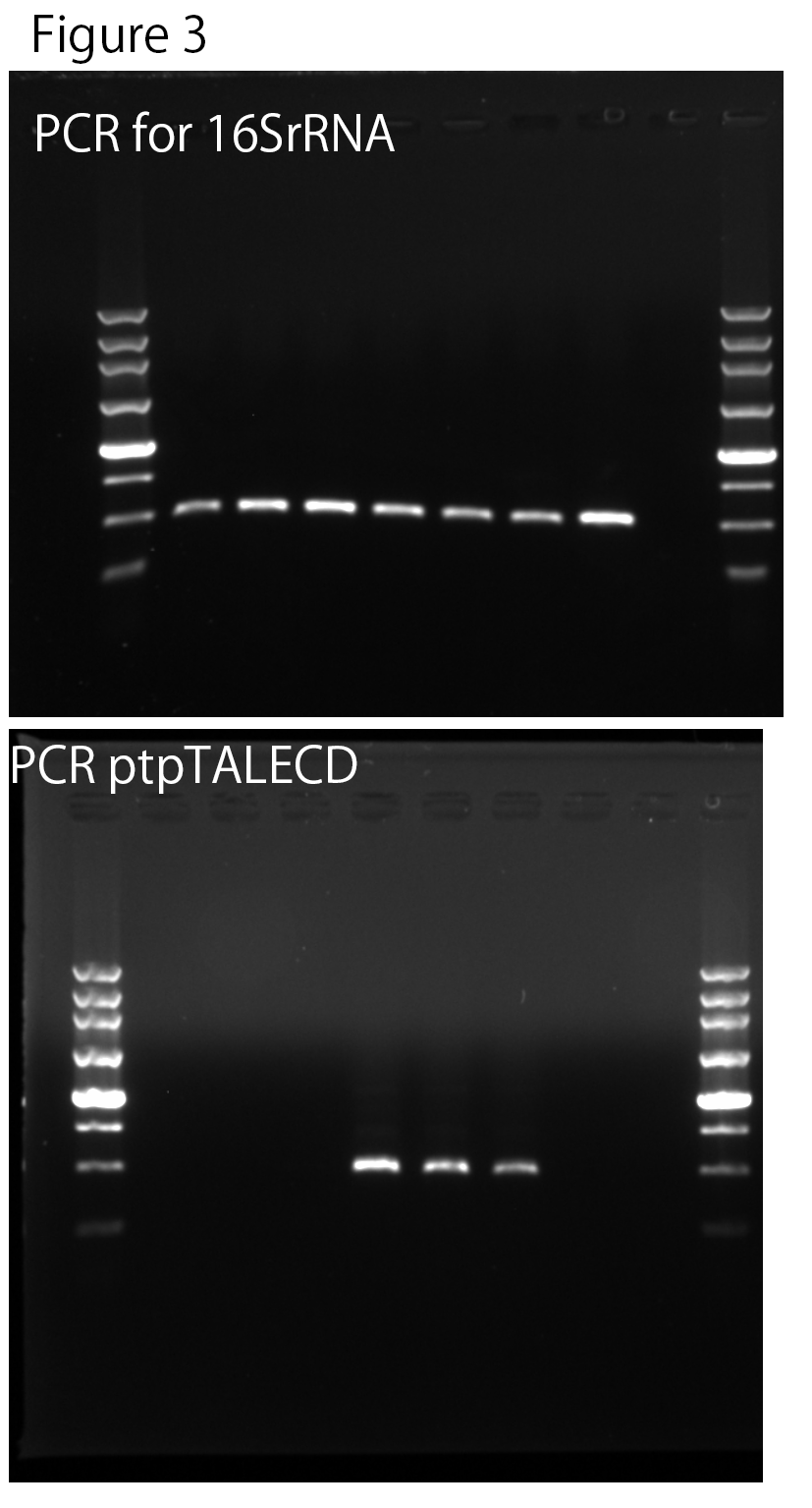

Supplement: Source Data Fig. 3 — (1) Unprocessed gel image (TALECD). (2) Unprocessed gel image (16S rRNA). [file 41477_2021_954_MOESM3_ESM.tif]
